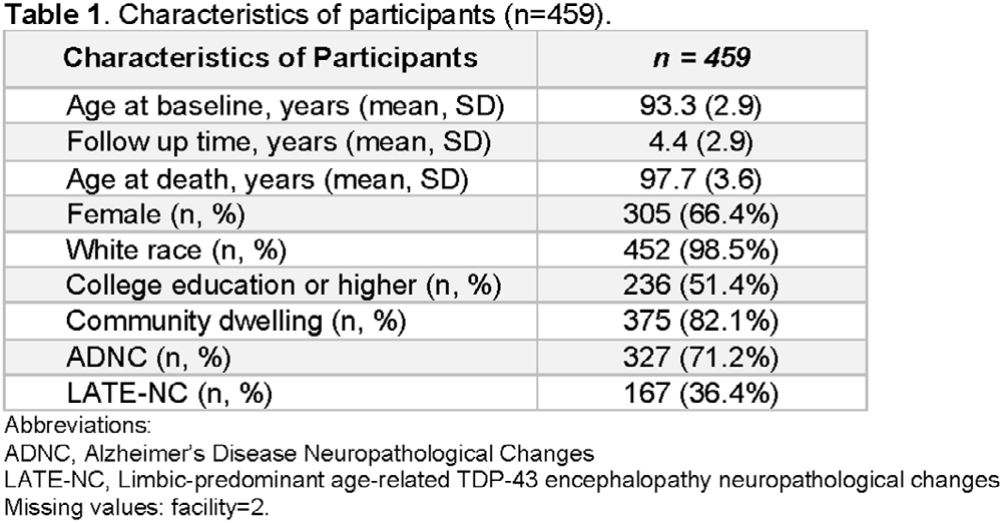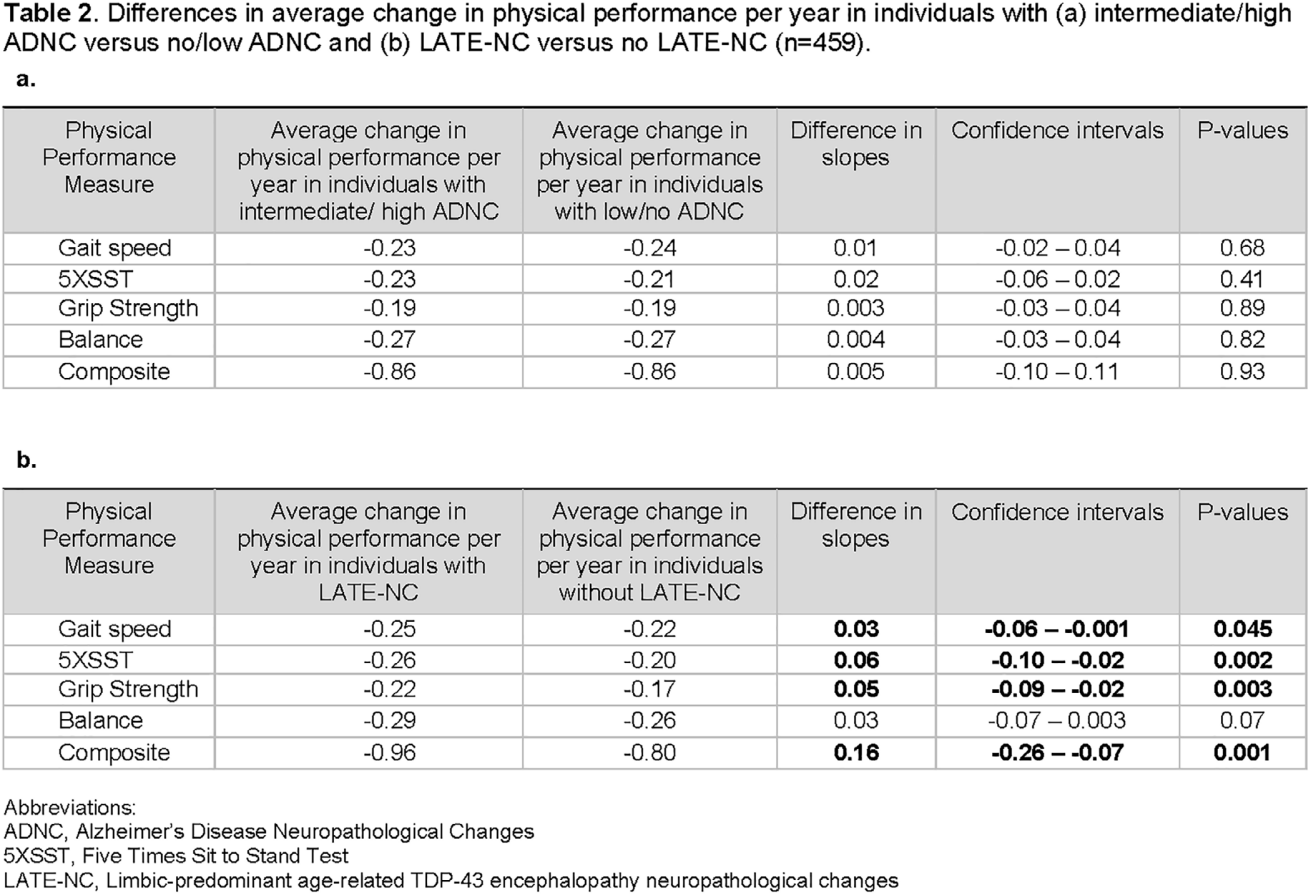# Limbic‐predominant age‐related TDP‐43 encephalopathy, but Not Alzheimer's Disease, neuropathological changes are associated with Physical Performance Decline in the Oldest Old: Insights from The 90+ Study

**DOI:** 10.1002/alz70855_106932

**Published:** 2025-12-24

**Authors:** Katherine Colcord, Luohua Jiang, Seyed Ahmad Sajjadi, Claudia H. Kawas, María M. M. Corrada

**Affiliations:** ^1^ University of California Irvine, Irvine, CA, USA; ^2^ Institute for Memory Impairments and Neurological Disorders (UCI‐MIND), University of California, Irvine, Irvine, CA, USA; ^3^ University of California, Irvine, Irvine, CA, USA

## Abstract

**Background:**

To examine physical performance longitudinal trajectories in relation to Alzheimer's Disease Neuropathological Changes (ADNC) and Limbic‐predominant age‐related TDP‐43 encephalopathy neuropathological changes (LATE‐NC) at autopsy. ADNC and LATE‐NC have similar cognitive presentations, but it is unclear whether physical presentation is similar as well.

**Method:**

Participants were from The 90+ Study, a longitudinal study of aging among individuals 90 years and older with evaluations every 6 months. We used 4 physical performance measures including gait speed, the Five Times Sit to Stand test (5XSST), grip strength, balance, and a composite summing the 4 measures. Each measure was scored from 0 (unable to perform) to 4 (best performance), the composite from 0 to 16. Neuropathological changes from brain autopsies were dichotomized: ADNC as intermediate/high versus none/low and LATE‐NC as present versus absent. To examine the longitudinal association of ADNC and LATE‐NC with physical performance trajectories, we used linear mixed regression models adjusted for sex, age at death, and education.

**Result:**

We analyzed data from 459 participants (305 women,154 men) with a mean age at death of 97.7 years (range 90‐111) and a mean follow up time of 4.4 years (range 0.1‐14) (Table 1). ADNC were not significantly associated with any physical performance measures. However, participants with LATE‐NC declined significantly faster in gait speed (difference in slopes=0.03 per year, *p* = 0.045), 5XSST (0.06 per year, *p* = 0.002), grip strength (0.05 per year, *p* = 0.003), and on the composite measure (0.16 per year, *p* = 0.001) compared to those without LATE‐NC (Table 2).

**Conclusion:**

LATE, but not AD, neuropathological changes are significantly associated with faster decline in physical performance in the oldest old. These findings indicate that, despite the similar cognitive presentations of LATE and AD, the physical manifestations of these neuropathological changes may be different. Further research into physical performance as a potential marker of LATE‐NC could improve our ability to distinguish between these neuropathological changes during life.